# The influence of iron on selected properties of synthetic pheomelanin

**DOI:** 10.1007/s12013-020-00918-1

**Published:** 2020-05-24

**Authors:** Andrzej Zadlo, Krystian Mokrzyński, Shosuke Ito, Kazumasa Wakamatsu, Tadeusz Sarna

**Affiliations:** 1 Department of Biophysics, Faculty of Biochemistry, Biophysics and Biotechnology, Jagiellonian University, Krakow, Poland; 2 Department of Chemistry, Fujita Health University School of Medical Sciences, Toyoake, Japan

**Keywords:** Pheomelanin, Iron, Transition metal ions, Photoprotection, Phototoxicity, Singlet oxygen

## Abstract

It is believed that while eumelanin plays photoprotective and antioxidant role in pigmented tissues, pheomelanin being more photoreactive could behave as a phototoxic agent. Although the metal ion-sequestering ability of melanin might be protective, transition metal ions present in natural melanins could affect their physicochemical properties. The aim of this research was to study iron binding by pheomelanin and analyze how such a binding affects selected properties of the melanin. Synthetic pheomelanin (CDM), prepared by enzymatic oxidation of DOPA in the presence of cysteine was analyzed by electron paramagnetic resonance (EPR) spectroscopy, spectrophotometry, chemical analysis, and time-resolved measurements of singlet oxygen phosphorescence. Iron broadened EPR signal of melanin and increased its optical absorption. Iron bound to melanin exhibited EPR signal at *g* = 4.3, typical for high-spin iron (III). Iron bound to melanin significantly altered the kinetics of melanin photodegradation, which in turn modified the accessibility and stability of the melanin–iron complexes as indicated by the release of iron from melanin induced by diethylenetriaminepentaacetic acid and KCN. Although bound to melanin iron little affects initial stages of photodegradation of CDM, the effect of iron becomes more pronounced at later stages of melanin photolysis.

## Introduction

Melanin is believed to play a photoprotective and antioxidant role in pigmented tissues [1–3]. This function is ascribed mainly to eumelanin. On the other hand, pheomelanin is more photoreactive and can exhibit phototoxic action [4]. In addition, photoinduced oxidative modifications of pheomelanin were shown to increase its potential to generate singlet oxygen and decrease its ability to quench this reactive oxygen species [5]. All natural melanins contain transition metal ions that may significantly modify observable properties of this polymer. Importantly, it was previously postulated that redox active transition metal ions may be involved in the etiology of skin melanoma [6]. Although the interaction of eumelanin with metal ions have previously been studied [7–14], there is little such information about pheomelanin, even though iron complex with pheomelanin was shown to increase UV-induced peroxidation of lipids [15]. Considering that most natural melanin pigments are actually a mixture of eumelanin and pheomelanin, and human epidermis melanin contains ~26% of pheomelanin, regardless of the degree of pigmentation [16], the effect of metal ions on photochemical properties of pheomelanin is an important issue. All natural melanins contain proteins and lipids which were well characterized in melanosomes from bovine eyes and in neuromelanin from human brains [17–19]. Synthetic melanin-protein conjugates are more similar to natural melanins than simple synthetic melanins. Recently, a new group of such soluble conjugates has been synthesized and the structure has been characterized. The iron binding was also characterized in these melanins. These melanin-protein conjugates were developed as models of human neuromelanin and they were able to activate microglia in cultures like human neuromelanin does [20]. However, the aim of this research was to study iron binding by pheomelanin and investigate the effect of iron on selected properties of the melanin. Considering that proteins are also able to bind iron, their presence would complicate the analysis of the photodegradation process. Therefore, in this study we used simple water-soluble synthetic model of pheomelanin (CDM), prepared by enzymatic oxidation of DOPA in the presence of cysteine.

## Materials and methods

### Reagents

l-β-(3,4-dihydroxyphenyl)alanine (l-DOPA), l-cysteine hydrochloride, mushroom tyrosinase, diethylenetriaminepentaacetic acid (DTPA), Chelex-100, and dialysis bags were from Sigma-Aldrich Chemie Gmbh (Steinheim, Germany). Potassium chloride, potassium dihydrogen phosphate, disodium hydrogen phosphate dodecahydrate, ferrous sulfate heptahydrate, glacial acetic acid and methanol were from Standard Co. (Lublin, Poland). Sodium chloride, potassium cyanide, 35–38% hydrochloric acid, 80% acetic acid, sodium hydroxide, and 98% sulfuric acid were from Polish Chemical reagents (POCH), (Gliwice, Poland).

All chemicals were reagent grade or better and used as supplied. Buffer solutions, made of water deionized by a millipore system (Millipore S A. 67120 Molsheim, France), were treated with Chelex-100 to remove traces of metal ions.

### Preparation of synthetic pheomelanin

Synthetic model of pheomelanin was prepared by enzymatic oxidation of DOPA in the presence of cysteine [21]. In brief, 0.789 g of l-DOPA and 1.06 g of l-cysteine hydrochloride were dissolved in 480 ml of 0.05 M phosphate buffer (pH 6.8) prepared from Na_2_HPO_4_ and KH_2_PO_4_. The solution was vigorously stirred until the amino acids dissolve. Overall 198,650 units of mushroom tyrosinase was dissolved in 20 ml of phosphate buffer and added to the solution of amino acids. The mixture was bubbled with oxygen at 25 °C for 4 h. After that the mixture was acidified by glacial acetic acid to pH 3 and stored on ice overnight. Then the mixture was centrifuged for 0.5 h at 5242 *g* at 4 °C. The precipitate was suspended in 200 ml of 1% acetic acid and centrifuged at the same conditions. Such washing procedure was carried out seven times. Finally, the precipitate was suspended in 21 ml of water. Such procedure yielded 0.108 g of CDM. Melanin was quantified by determination of its dry mass.

### Preparation of iron complexes with pheomelanin and citrate

Iron complex with pheomelanin was prepared similarly as previously described for eumelanin [12]. Because iron (II) salts undergo hydrolysis above pH 5.8, ferrous sulfate was dissolved in 10^−4^ M H_2_SO_4_ and melanin was adjusted to pH 5. To prevent oxidation of iron (II), 10^−4^ M H_2_SO_4_ was saturated with argon. Overall 4 mg/ml melanin was mixed with water and 0.025 M ferrous sulfate so that the final concentration of melanin was 2 mg/ml and iron 0.358 mM i.e., 1% (w/w). After 0.5 h incubation the pH was adjusted to 7.4 and further incubated at room temperature in dark. Control melanin was prepared similarly except the addition of ferrous sulfate.

Iron complex with citrate was prepared using iron (III) as previously described [14]. In brief, 0.1 M citric acid was added to 0.025 M ferric chloride in 0.02 M hydrochloric acid. Under such conditions, citrate: metal molar ratio was 2:1. Then the pH was adjusted to 7 by drop-wise addition of 1 M NaOH. Finally, water was added, adjusting the metal ion concentration to 10 mM.

### EPR spectroscopy

All EPR measurements, except these at 20 K, were performed using Bruker EMX-AA EPR spectrometer (Bruker BioSpin, Rheinstetten, Germany). EPR spectroscopy at 77 K was used to examine the influence of iron on EPR signal of pheomelanin, to monitor progress of melanin photodegradation [10, 22] and to record iron EPR signal. These measurements were carried out at melanin concentration 1 mg/ml in PBS (pH 7.4), in 45 mM DTPA adjusted to pH 7.4 or in 45 mM DTPA with 5 mM KCN. At pH 7.4 DTPA exhibited weak buffer capacity and addition of KCN increased the pH only to 7.8. Typical instrumental settings for measurements of melanin were modulation amplitude, 0.305 mT; center field, 336.2 mT; scan range, 7 mT; scan time, 42 s; time constant, 327.7 µs and microwave power, 32.4 µW. The final EPR spectra of melanin were taken from averaging 10 scans. Iron was measured at following conditions: modulation amplitude, 0.805 mT; center field, 158.49 mT; scan range, 100 mT; scan time, 84 s; time constant, 327.7 µs and microwave power, 5.29 mW. The final EPR spectra of iron were averages of 5 scans.

**Fig. 1:**
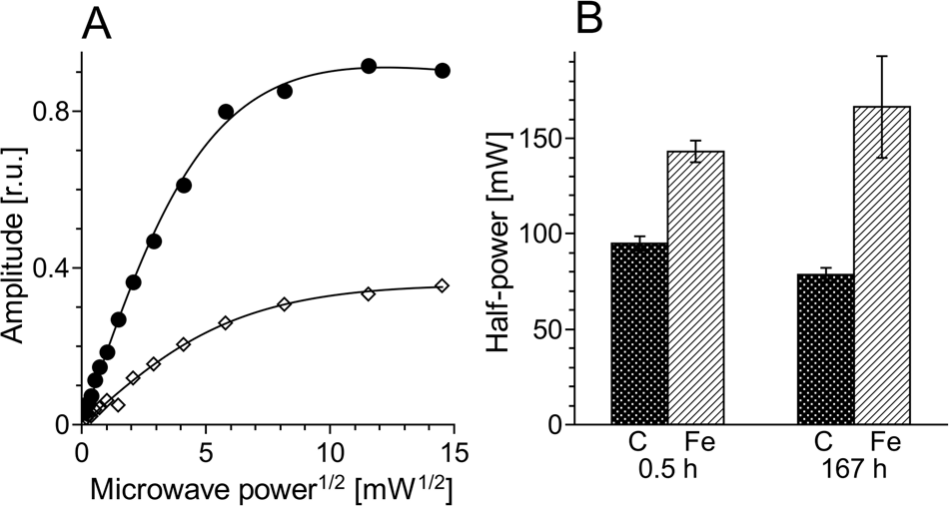
The effect of iron on room temperature EPR signal of 2 mg/ml CDM. **a** Melanin signal amplitude plotted against square root of microwave power and fitted with the following function: *f*(*x*) = *A*·*x*·[1 + (2^1/ε^ – 1)·*x*^2^/P_1/2_]^–ε^ [23] as described in “Materials and methods” section. Filled circles—control melanin without iron, open diamonds—melanin with 1% (w/w) iron. **b** Half power in samples containing 2 mg/ml CDM without (C) and with (Fe) 1% (w/w) iron after different incubation time

The efficiency of melanin–iron interaction was determined by measurements of the influence of iron on microwave power saturation of the EPR signal of melanin as described in Zadlo et al. [14]. Such measurements were carried out at room temperature at melanin concentration 2 mg/ml in water without buffer using EPR flat cell (0.3 mm thickness and 8 mm width). Just before EPR measurements, the sample pH was adjusted to 7.4. Typical instrumental settings were: modulation amplitude 0.305 mT; center field 339.2 mT; scan range 5 mT; scan time 42 s and time constant 327.7 µs. Microwave power was selected in the range 0.00839–211 mW. EPR signal amplitude was plotted against square root of microwave power (Fig. 1a) and half power (*P*_1/2_) was determined from fit of equation:

*f*(*x*) = *A*·*x*·[1 + (2^1/ε^ – 1)·*x*^2^/*P*_1/2_]^–ε^ [23], where *x* is a square root of microwave power, *f*(*x*) is EPR signal amplitude, *A* is initial slope, ε is homogeneity coefficient, and *P*_1/2_ is half power i.e., microwave power, at which the first derivative amplitude is reduced to half of its unsaturated value.

The sample of CDM with 1% (w/w) iron was also measured at 20 K. Such measurements were carried out using Bruker ELEXYS spectrometer (Bruker BioSpin, Rheinstetten, Germany) equipped with Oxford Instruments cryogenic system. The concentration of CDM was 2 mg/ml. Instrumental settings were: modulation amplitude, 1.6 mT; center field, 250 mT; scan range, 300 mT; scan time, 167.8 s; time constant, 327.7 µs, and microwave power, 5.12 mW.

### Photodegradation of pheomelanin

CDM with and without iron was diluted to 1 mg/ml in water, adjusted to pH 7.4 and irradiated with 400 nm (265 mW/cm^2^) light originating from 100 W diode array illuminator (High Power UV Purple LED Chip, Chanzon, China). The initial volume of melanin solution subjected to photodegradation was 5 ml, the inner diameter of the vessel was 4.7 cm and the whole surface of solution was irradiated. During degradation, the samples were gently stirred. Every several hours, the pH was corrected to 7.4 by addition of NaOH solution. At selected time intervals, measured amount of melanin solution was withdrawn for EPR spectroscopy, measurements of optical absorption and chemical analysis of melanin subunits. Before taking each melanin sample, the vessel with reaction mixture was weighted and the volume was corrected by the addition of water.

### Measurements of optical absorption of melanin

Optical absorption of melanin was measured in 1 M NaOH as previously described [12]. In brief, 26.3 µl aliquot of sample containing 1 mg/ml melanin was added to 0.5 ml of NaOH, shaken on Vortex shaker and the optical absorption was measured 1 min after addition of melanin to NaOH. To reduce light-scattering, the measured absorbance at 800 nm was subtracted from all values of absorbance at other wavelengths. Optical absorption was integrated in the spectral range 350–550 nm.

### Time-resolved singlet oxygen detection

D_2_O solutions of CDM samples in a 10-mm-optical path quartz fluorescence cuvette (QA-1000; Hellma, Mullheim, Germany) were excited by monochromatic light pulses generated by an integrated nanosecond DSS Nd:YAG laser system equipped with a narrow-bandwidth optical parameter oscillator (NT242-1k-SH/SFG; Ekspla, Vilnius, Lithuania). The light pulses were delivered at repetition rate 1 kHz; their energy was tens of microjoules in the UVA–UVB spectral region and up to several hundred microjoules in the visible region. Photoexcitation of CDM melanin samples in D_2_O was examined in 320–600 nm spectral range. Singlet oxygen phosphorescence was measured perpendicularly to the excitation beam in a photon-counting mode using a thermoelectric cooled NIR PMT module (H10330-45, Hamamatsu, Japan) equipped with a 1100 nm cutoff filter and additional dichroic narrow-band filter NBP, selectable from the spectral region range of 1150–1355 nm (NDC Infrared Engineering Ltd, Bates Road, Maldon, Essex, UK). Data were collected using a computer-mounted PCI-board multichannel scaler (NanoHarp 250; PicoQuant GmbH, Berlin, Germany). Data analysis, including first-order luminescence decay fitted by the Levenberg–Marquardt algorithm, was performed by custom-written software. The acquisition time for obtaining singlet oxygen phosphorescence signals was 30 s.

Rose Bengal dissolved in D_2_O (absorbance in 500 nm ~0.11), used as a photosensitizer in experiments designed to determine rate constants of singlet oxygen quenching by CDM melanin samples, was excited with 550-nm laser pulses, attenuated with three pieces of wire mesh (light transmission of each piece ~30%) to adjust photoexcitation energy. To determine quantum yield of singlet oxygen photogeneration by CDM melanins, fluorescein, and scopoletin were used as references for 425 nm and 365 nm, respectively. Quantum yield of singlet oxygen photogeneration by fluorescein was determined (using rose Bengal as a reference) upon excitation at 500 nm (absorbance of both was 0.108 ± 0.002), whilst quantum yield of singlet oxygen photogeneration by scopoletin was determined (using fluorescein as reference) upon excitation at 327 nm (absorbance of both was 0.145 ± 0.003). Both laser pulses were attenuated by application of appropriate filters. All dyes were dissolved in D_2_O. Quantum yields of singlet oxygen photogeneration by CDM melanin samples were determined by comparative measurements of the initial intensities of 1270-nm phosphorescence emitted by fluorescein (for 425-nm quantum yields), scopoletin (for 365-nm quantum yields), and CDM melanins excited with laser pulses of increasing energies. Fluorescein and melanins were dissolved in D_2_O and their absorbance at 425 nm was adjusted to 0.125. Scopoletin and melanins were dissolved in D_2_O and their absorbance was adjusted to 0.134. All samples were constantly mixed during measurement process using a magnetic stirrer.

### Chemical analysis of melanin subunits

CDM samples without and with iron were subjected to reductive hydroiodic hydrolysis [24, 25] and alkaline hydrogen peroxide oxidation [26].

## Results

**Fig. 2:**
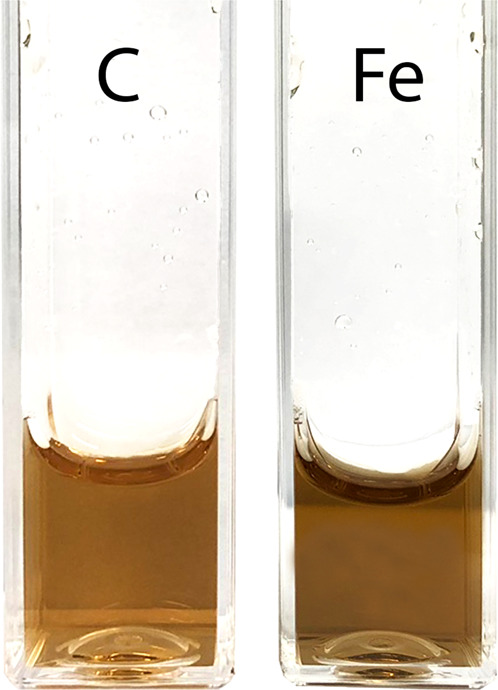
Photograph of 0.1 mg/ml water solutions of CDM without (C) and with (Fe) 1% (w/w) iron

**Fig. 3:**
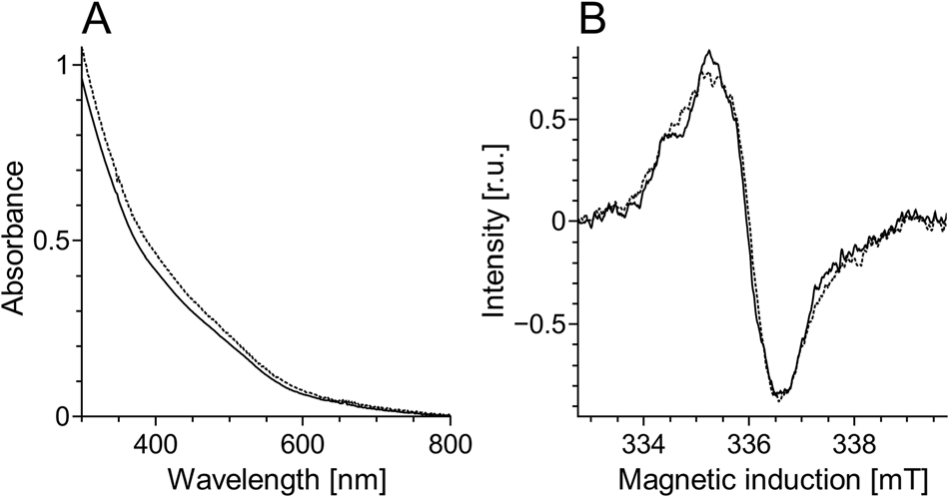
UV–vis spectra of CDM diluted to 0.05 mg/ml in 1 M NaOH (**a**) and EPR spectra of 0.5 mg/ml CDM in 1 M HCl (**b**). Continuous line—CDM without iron, dotted line—CDM with 1% (w/w) iron

**Fig. 4:**
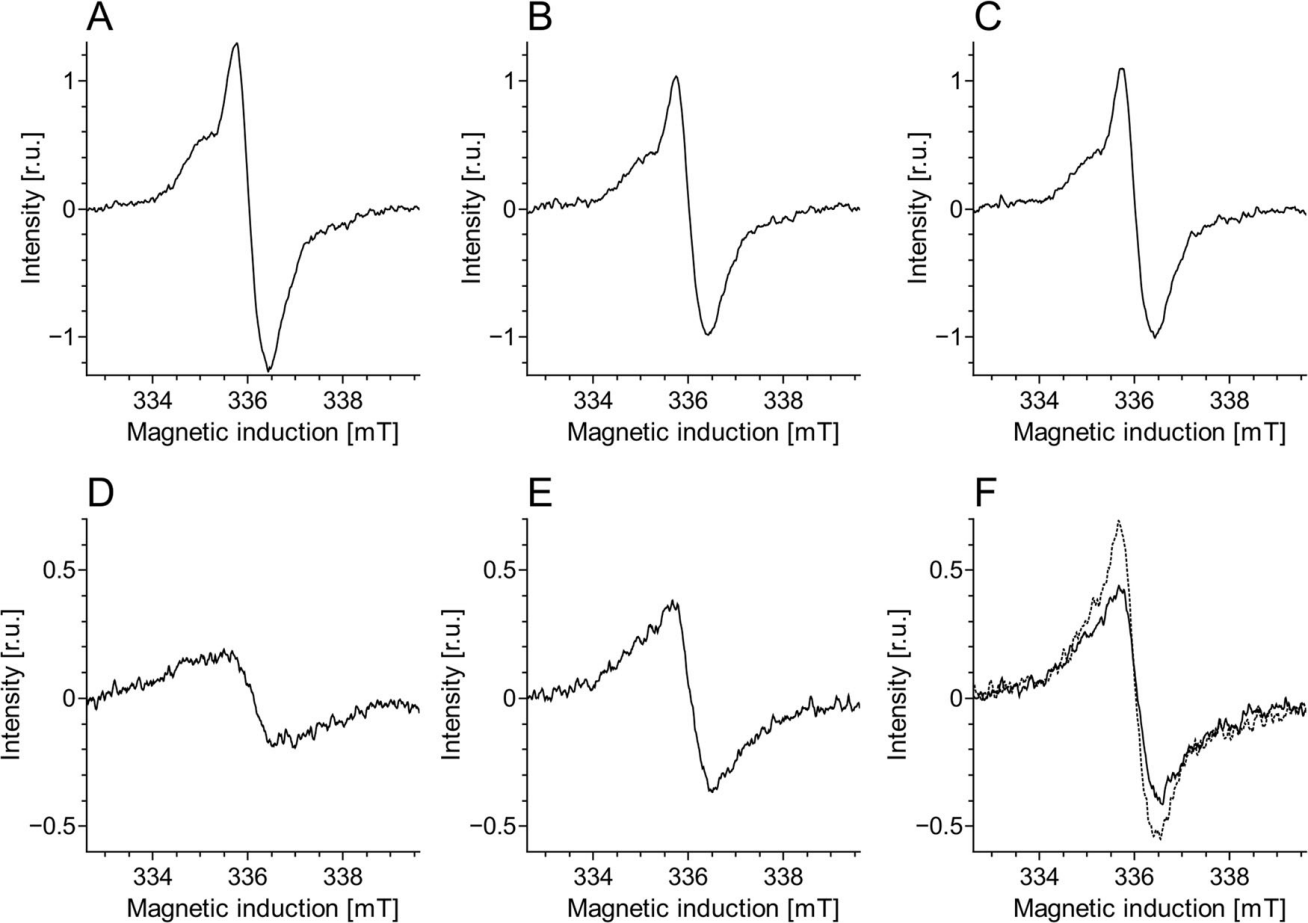
7 K EPR spectra of CDM without (**a**–**c**) or with (**d**–**f**) 1% (w/w) iron. These EPR spectra were registered after dilution of CDM to 1 mg/ml in PBS, pH 7.4 (**a**, **d**), 45 mM DTPA adjusted to pH 7.4 (**b**, **e**) or 45 mM DTPA with 5 mM KCN (resultant pH 7.8) (**c**, **f**) and their incubation at room temperature for 1 h (continuous line) or 24 h (dotted line)

**Fig. 5:**
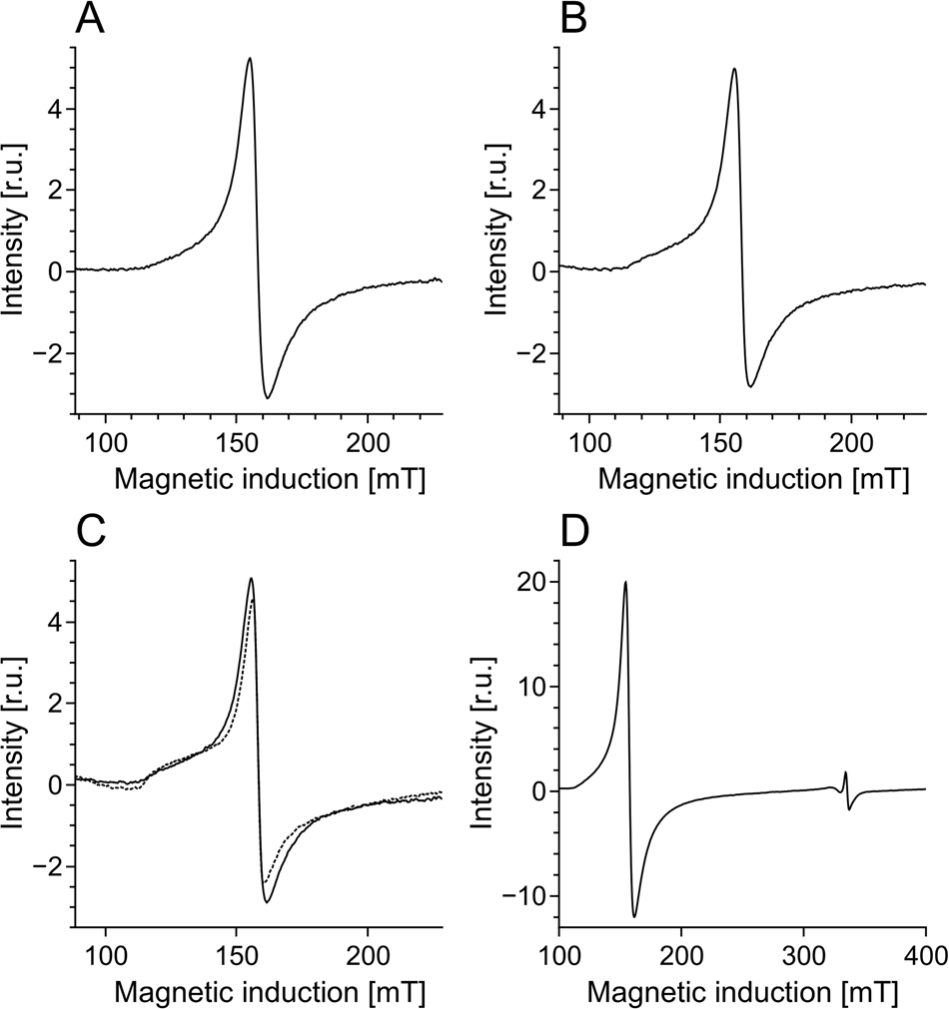
EPR spectra of iron bound to CDM. EPR spectra **a**–**c** were registered at 77 K after dilution of CDM to 1 mg/ml in PBS, pH 7.4 (**a**), 45 mM DTPA adjusted to pH 7.4 (**b**), or 45 mM DTPA with 5 mM KCN (resultant pH 7.8) (**c**) and their incubation at room temperature for 1 h (continuous line) or 24 h (dotted line). **d** Wide scan carried out at 20 K at CDM concentration 2 mg/ml

**Fig. 6:**
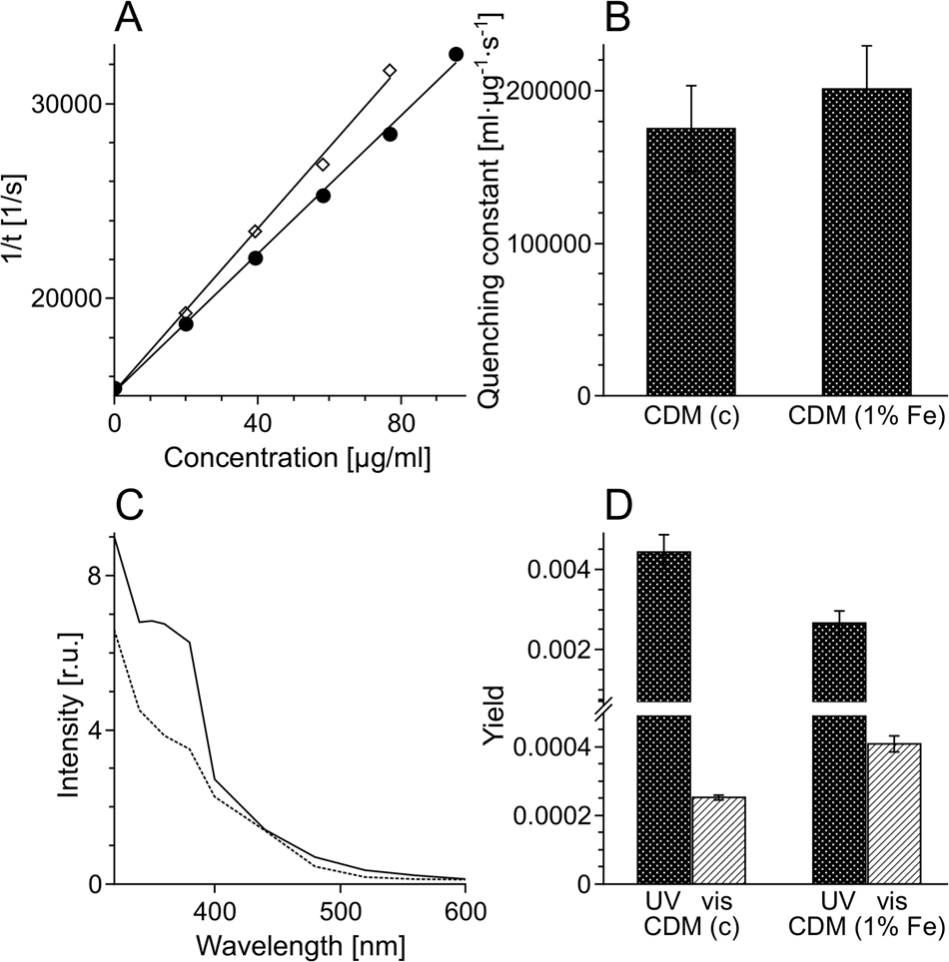
The effect of iron on effectiveness of quenching of singlet oxygen and on the yield of photoinduced generation of this reactive oxygen species. **a** Inverse of singlet oxygen lifetime plotted against the concentration of CDM without (filled circles) and with (open diamonds) 1% (w/w) iron. **b** Constant of quenching of singlet oxygen. **c** Action spectra of photoinduced formation of singlet oxygen by CDM without iron (continuous line) or with 1% (w/w) iron (dotted line). **d** Yield of genergbation of singlet oxygen by CDM excited with 365 nm (UV) or 425 nm (vis) light

**Fig. 7:**
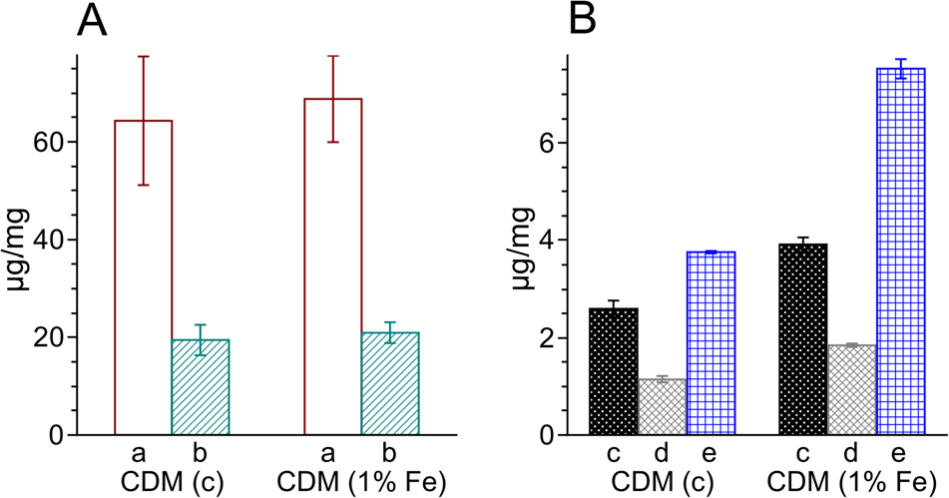
Results of chemical analysis of melanin subunits. **a** Analysis by HI hydrolysis: bar a—4-AHP, bar b—3-AHP. **b** Analysis by alkaline H_2_O_2_ oxidation: bar c—PTCA, bar d—PDCA, bar e—TTCA. The presented values are averages from two analyses

**Fig. 8:**
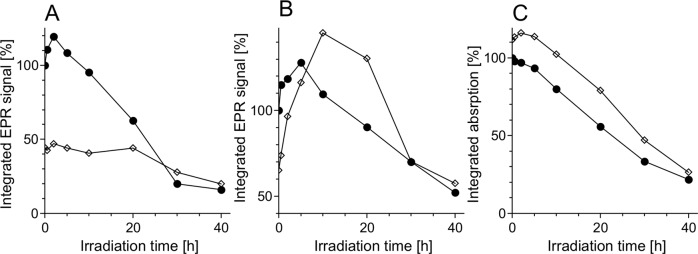
Time-dependent changes of 77 K integrated EPR signal of CDM (**a**, **b**) and 350–550 nm integrated optical absorption (**c**) irradiated with 400 nm (265 mW/cm^2^) light. EPR measurements were carried out in PBS, pH 7.4 (**a**) or in 45 mM DTPA with 5 mM KCN, pH 7.8 (**b**). Optical absorption was measured in 1 M NaOH. Filled circles—control melanin without iron, open diamonds—melanin with 1% (w/w) iron

**Fig. 9:**
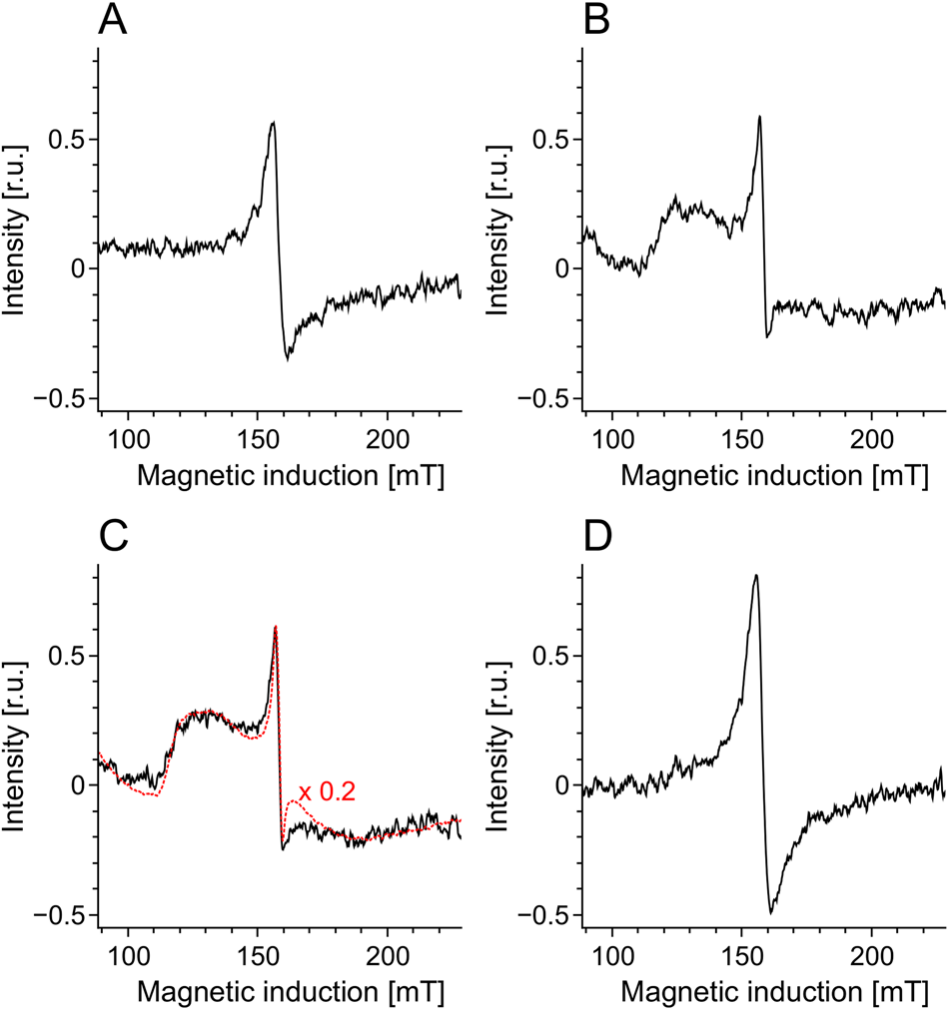
77 K EPR spectra of iron bound to CDM photodegraded for 40 h except dotted line in **c**. EPR measurements were carried out after the dilution of CDM samples to 0.5 mg/ml in PBS, pH 7.4 (**a**), 45 mM DTPA with 5 mM KCN, pH 7.8 (**b**), 45 mM DTPA, pH 7.4 (**c**), water, pH 7.4 (**d**). Dotted line in **c**—0.1791 iron complex with citrate after addition of 45 mM DTPA. EPR signal of iron in citrate—DTPA system is multiplied by 0.2

Half power (*P*_1/2_) of CDM without iron was determined to be about 90 mW (Fig. 1b). Iron strongly increased the value of *P*_1/2_ and the effect increased with the time of incubation of CDM with iron. CDM with iron was visually darker (Fig. 2) and exhibited increased optical absorption (Fig. 3a). Iron-induced optical changes were not accompanied with significant changes of EPR signal of melanin recorded at low pH when iron was released (Fig. 3b). On the other hand, iron strongly broadened EPR signal of CDM and decreased its amplitude measured at pH 7.4 (Fig. 4a, d). Although 1 h incubation of iron-containing CDM with DTPA or KCN caused some increase of the amplitude of melanin EPR signal (Fig. 4e, f), comparison with CDM without iron measured under the same conditions (Fig. 4b, c) indicates that the effect of these strongly binding ligands was rather weak. In addition, DTPA and KCN had negligible effect on EPR signal of iron bound to melanin (Fig. 5a–c). On the other hand, 24 h incubation with these ligands caused substantial increase of EPR signal of melanin (Fig. 4f) and significant alterations in iron EPR signal (Fig. 5c). A wide scan at 20 K did not reveal any other iron signal except the high-spin iron (III) signal (Fig. 5d). CDM with iron quenched singlet oxygen more efficiently than control without iron (Fig. 6a, b); however, the effect was statistically insignificant. Although iron decreased UV-induced formation of singlet oxygen (Fig. 6c, d), the yield of blue light-induced generation of singlet oxygen was increased by a factor of 1.6 (Fig. 6d). Chemical analysis of CDM showed that the content of 4-amino‐3‐hydroxyphenylalanine (4-AHP) and 3-amino‐4‐hydroxyphenylalanine (3-AHP), which are degradative markers of benzothiazine units characteristic for pheomelanin [24] were not affected by iron (Fig. 7a). Interestingly, iron slightly increased the content of pyrrole-2,3-dicarboxylic acid (PDCA) and pyrrole-2,3,5-tricarboxylic acid (PTCA) (Fig. 7b). PDCA and PTCA are degradative markers of 5,6-dihydroxyindole (DHI) and 5,6-dihydroxyindole-2-carboxylic acid (DHICA) units of eumelanin, respectively [26], but in the case of CDM, which is expected to be almost pure pheomelanin, the effect seems to be nonspecific. In addition, iron increased the content of thiazole-2,4,5-tricarboxylic acid (TTCA) by a factor of 2, which is a degradative marker derived from benzothiazole unit that accumulates in oxidatively modified pheomelanin [5]. Irradiation of 1 mg/ml CDM with 400 nm (265 mW/cm^2^) light caused gradual decrease of EPR signal of melanin (Fig. 8a, b) and integrated optical absorption (Fig. 8c). The decrease of EPR signal was preceded by transient increase of this signal. Although iron seemed to inhibit initial decrease of optical absorption of CDM, it accelerated such optical changes of melanin at later stages of its photodegradation. Addition of DTPA and KCN to strongly degraded CDM with iron caused strong alteration of iron EPR signal (Fig. 9b, c). EPR signal of iron in the sample with such degraded CDM in the presence of DTPA was similar to EPR signal of iron with DTPA in the absence of melanin, although it was five times weaker (Fig. 9c). At this degradation stage, even addition of phosphate buffer caused a decrease of iron EPR signal (Fig. 9a, d).

## Discussion

Comparison with our previous studies [14] indicates that EPR signal of pheomelanin without iron saturated at higher microwave power (Fig. 1) than corresponding eumelanin. The same studies indicate that room temperature *P*_1/2_ is a good indicator of iron binding by melanin [14]. Thus significantly higher *P*_1/2_ of iron-containing CDM than that of CDM without iron (Fig. 1b) indicates that iron is efficiently bound by pheomelanin. The binding of iron by CDM is also indicated by a strong decrease of the amplitude of the EPR signal of melanin, which coincides with the signal broadening clearly observed at 77 K (Fig. 4a, d). These effects of iron on EPR signal of CDM result from the efficient dipole–dipole interaction of the melanin radicals with paramagnetic iron ions bound to melanin [27]. Longer incubation time of melanin with iron ions increases the efficiency of the dipolar interaction, which is reflected by further increase of *P*_1/2_ (Fig. 1b). It appears that iron incubated with CDM for a long time (about one weak) is bound by subunits that are localized deeper in the melanin polymer. This conclusion is supported by a weak effect of DTPA and KCN on EPR signal of CDM-Fe(III) (Fig. 4d–f). Consistently with our conclusion, short-time incubation of CDM with these strongly binding ligands had almost no effect on EPR signal of iron bound to melanin (Fig. 5), which indicates that iron bound to CDM is mostly inaccessible for reagents that are unable to penetrate into melanin polymer. Such weak effect has steric rather than thermodynamic explanation. Even partial removal of iron from neuromelanin with a strong chelator like deferoxamine takes up to 24 h [28]. Incubation of CDM with DTPA and KCN for 24 h caused partial removal of iron from the melanin, indicating that this process also is thermodynamically feasible. It is important to stress that when iron (II) was added to CDM, the EPR signal of iron bound to CDM was a slightly asymmetric, single line at *g* = 4.3 (Fig. 5a), typical for high-spin iron (III) complex with melanin [8]. This indicates that binding of iron (II) by pheomelanin is accompanied by its rapid and efficient oxidation. Such phenomenon was previously observed in the case of synthetic eumelanin [14]. Parameters of EPR signal of iron bound to CDM suggest that iron is complexed by pheomelanin in similar manner as in the case of eumelanin. However, the signal intensity was 1.5 times lower than that registered at the same conditions for EPR signal of iron bound to eumelanin [14]. Apart from pheomelanin having a lower iron-binding capacity than eumelanin, another explanation is that pheomelanin may contain a fraction of iron in another form than high-spin iron (III). However, EPR measurements at 20 K did not show any other form of iron ions bound to melanin, such as low-spin iron (III) and only high-spin iron (III) was detected (Fig. 5d). Another form of iron that, in principle, could also form complexes with CDM is high-spin or low-spin iron (II). Although the former is practically undetectable by conventional EPR spectroscopy and the latter is diamagnetic, Mössbauer spectroscopy of substantia nigra showed very low (no more than 5%) content of iron (II) [29]. In addition to mononuclear high-spin Fe(III) bound to catechols, there could be polynuclear Fe(III) clusters bound by oxy-hydroxy bridges as shown with other Mössbauer spectroscopy study [30, 31]. It is important to stress that Galazka-Friedman et al. suggested the dominant role of ferritin in binding of iron in substantia nigra [29]. Nevertheless, our recent study showed that iron complexed with eumelanin occurs at +3 oxidation state [14]. Iron-induced darkening of CDM (Fig. 2) and the increase of its optical absorption (Fig. 3) is accompanied by slight increase of PDCA and PTCA (Fig. 7a) (considered to be markers of DHI and DHICA units characteristic for eumelanin) [26] and pronounced increase of TTCA (Fig. 7b) (a marker of modified benzothiazole units formed by oxidative degradation of pheomelanin [5]). Although the observed increase of PDCA and PTCA is not a totally reliable indicator of changes in the content of eumelanin, significant increase of TTCA suggests iron-catalyzed oxidation of CDM. The conversion of benzothiazine to benzothiazole is known to be accompanied by the modification of pheomelanin structure by heat or light [26, 32, 33]. Our previous studies showed that light-induced oxidative modification of pheomelanin caused both increase of modified benzothiazole and decrease of benzothiazine [5]. However, iron-induced increase of TTCA was not accompanied with the decrease of 4-AHP (Fig. 7a) a characteristic indicator of benzothiazine units [24]. It indicates that iron does not cause degradation of CDM benzothiazine units. According to our previous study, chemical analysis of pheomelanin subjected to photodegradation showed a decreased content of native benzothiazole units but an increase of TTCA [5], suggesting TTCA were derived from oxidatively modified benzothiazole. Therefore, it appears that iron-catalyzed oxidation of melanin subunits is different than the light-induced process.

The influence of iron on the efficiency of CDM to quench singlet oxygen is too weak to draw any conclusions (Fig. 6a, b). On the other hand it is of interest that iron ions seem to modify the efficiency of CDM to photogenerate singlet oxygen differently, depending on the exciting light spectral region (Fig. 6c, d). Although different factors could be involved, the data suggest iron-induced chemical modification of specific melanin subunits rather than aggregation.

Light-induced decrease of EPR signal and optical absorption of CDM indicate the progress of degradation of melanin polymer (Fig. 8). Transient increase of EPR signal at the beginning of irradiation, that is visible especially in the case of CDM without iron (Fig. 8a) results from reversible oxidation of the melanin subunits [9, 10]. In the case of iron-containing CDM, the effect of transient oxidation at the beginning of irradiation is well reflected in the initial changes of optical absorption (Fig. 8c). Due to strong influence of iron on the EPR signal of melanin (Figs. 4a, d and 8a) the measurements of integrated optical absorption are the most reliable method to compare the progress of photodegradation of CDM without and with iron (Fig. 8c). Lower initial rate of light-induced decrease of optical absorption of iron-containing CDM in comparison to CDM without iron may be an effect of transient melanin oxidation. On the other hand, the acceleration of photodegradation of CDM with iron at later stages of the melanin irradiation (Fig. 8c) may result from increased exposure of bound to melanin iron to the external environment. This conclusion is supported by the kinetics of changes of EPR signal of iron-containing CDM measured in the presence of DTPA and KCN (Fig. 8b). This kinetics is a superposition of degradation of melanin and loss of iron that becomes accessible for strong low-molecular ligands present in the external environment. DTPA and KCN-induced strong changes of the EPR signal of iron bound to CDM degraded for 40 h (Fig. 9b, c) confirm that iron in the degraded CDM is accessible for these strongly binding ligands. Although CN^−^ anion is much smaller, the difference between the spectra in Fig. 9b, c is insignificant which indicates that iron is accessible even for high molecular weight ligands. After addition of DTPA to degraded iron-containing CDM, the EPR signal of iron was similar to that registered after addition of DTPA to iron complex with citrate, although it was five times weaker (Fig. 9c). Thus the observed EPR signal is not due to iron complexes with melanin, it corresponds to iron ions released from degraded CDM that were intercepted by DTPA. Weaker EPR signal of iron in system with degraded CDM than in citrate system indicates that some iron underwent hydrolysis and precipitated as iron hydroxide during photodegradation. Therefore iron ions can be intercepted from strongly degraded CDM even by hydroxide anion. The EPR signal of iron in PBS (Fig. 9a) is weaker than the iron(III) spectrum recorded in water (Fig. 9d), which indicates that phosphate anions from PBS are able to precipitate part of iron from such degraded melanin. It is important to stress that iron-induced formation of modified benzothiazole may be an additional factor responsible for accelerated late-stage degradation of iron-containing CDM. Such benzothiazole was previously shown to be responsible for increased photoreactivity of photobleached pheomelanin [5].

In conclusion, iron is efficiently bound by pheomelanin and binding of iron (II) is accompanied by its oxidation.

Iron catalyzes oxidation of pheomelanin and thus increases the presence of modified benzothiazole.

Most of iron bound to non-degraded pheomelanin is localized inside the polymer and is inaccessible for external environment. On the other hand, photodegradation of pheomelanin increases exposure of iron, which together with elevated content of modified benzothiazole can increase susceptibility of pheomelanin to further photodegradation and elevated photoreactivity and phototoxicity of such melanin. If similar processes take place in human skin they could be responsible for light and redox active metal ion-induced increase of phototoxicity of pheomelanin, which might be involved in the etiology of skin melanoma.
